# Effects of chitosan-assisted moderate-intensity exercise on metabolic parameters and lower-limb biomechanical characteristics in overweight adults: a randomized controlled trial

**DOI:** 10.3389/fbioe.2025.1629042

**Published:** 2025-09-19

**Authors:** Jianwei Zhang, Haiyan Liu

**Affiliations:** ^1^ College of Health, Zhejiang Industry Polytechnic College, Shaoxing, Zhejiang, China; ^2^ College of Huangjiu, Zhejiang Industry Polytechnic College, Shaoxing, Zhejiang, China

**Keywords:** overweight individuals, polysaccharides, exercise intervention, biomechanical, indices

## Abstract

**Background:**

Overweight is a major risk factor for metabolic disorders and joint injuries. Exercise and dieting alone often fail to yield ideal outcomes due to issues like non-compliance and safety concerns. Polysaccharides, such as chitosan, are promising in regulating lipids, glucose, and aiding weight loss. However, the combined effects of chitosan supplementation and exercise on lower limb biomechanics and metabolic health remain underexplored.

**Objective:**

To assess the effects of chitosan supplementation combined with moderate-intensity exercise on metabolic parameters and lower limb biomechanics in overweight adults, and to evaluate the intervention’s safety, compliance, and feasibility for clinical and community-based management.

**Methods:**

A single-center, parallel-group, randomized controlled trial (RCT) was conducted with 60 overweight adults. Participants were randomly assigned to one of three groups: polysaccharide plus exercise, exercise-only, or control group, with a 12-week intervention period. The polysaccharide plus exercise group received 3,000 mg of daily chitosan alongside moderate-intensity exercise, while the exercise-only group participated in the same exercise routine without supplementation. The control group maintained their usual lifestyle. Primary outcomes included changes in body composition, metabolic indices, and lower limb biomechanics. Compliance and adverse events were recorded. Data were analyzed using one-way ANOVA, repeated-measures ANOVA, and Bonferroni correction (P < 0.05).

**Results:**

No significant baseline differences were found across groups (P > 0.05). After 12 weeks, the polysaccharide plus exercise group showed significantly greater reductions in body weight and waist circumference compared to the exercise-only and control groups (P < 0.05). BMI, hip circumference, and total body fat mass were also significantly lower than the control group (P < 0.05). The polysaccharide plus exercise group exhibited superior improvements in HOMA-IR, total cholesterol, triglycerides, LDL, and HDL (P < 0.05), and showed more substantial reductions in maximum plantar pressure and peak ground reaction force (P < 0.05). Compliance in this group was significantly higher than in the exercise-only group (P = 0.002). No significant differences in adverse events were observed (P > 0.05).

**Conclusion:**

Chitosan supplementation combined with moderate-intensity exercise significantly improved body weight, metabolic parameters, and lower limb biomechanics in overweight individuals, with good compliance and safety. This intervention offers a promising strategy for managing overweight and related metabolic issues.

## 1 Introduction

The global prevalence of overweight and obesity continues to rise, having become a major factor threatening public health. Recent WHO estimates indicate that in 2022, 2.5 billion adults (43%) were overweight, including 890 million living with obesity (16%), and adult obesity has more than doubled since 1990 (World Health Organization, 2025). Research shows that overweight status is often accompanied by insulin resistance, dyslipidemia, and other metabolic disorders, and is closely related to the occurrence of joint degeneration and musculoskeletal system diseases (Zhang et al., 2025). Elevated body mass index and circumferential measurements are often neglected in young and middle-aged populations, but may subsequently develop into serious chronic diseases (Chen et al., 2025). In order to encourage earlier intervention in overweight populations and obtain safe, feasible weight management plans, more pragmatic research is needed at both clinical and community levels. Diet control and exercise intervention are common weight loss strategies, but some patients fail to achieve stable effects due to lifestyle or adherence issues (Rabi Bazaz et al., 2025; Santisteban et al., 2024). Polysaccharides have shown potential in regulating blood glucose and lipids, improving intestinal microecology, and reducing fat absorption, among which chitosan has attracted widespread attention due to its high safety and tolerance (Feizolahi et al., 2024). With the help of systematic exercise prescriptions, polysaccharides may enhance metabolic efficiency and synergistically reduce cardiovascular and bone-joint burdens (Bajaber et al., 2024; Wong et al., 2024). However, the actual effectiveness and feasibility of polysaccharide-exercise synergistic interventions need to be verified by more rigorous research designs. Existing literature has reported certain improvements in metabolic indicators such as blood glucose and lipids under polysaccharide interventions (Nadhir et al., 2023), but there is a lack of in-depth discussion on their effects on lower limb biomechanical characteristics and exercise function. Clinically, overweight populations often face lower limb joint pain and structural damage as they age, while simplified or blind exercise plans may increase joint burdens (Lebenstei et al., 2023; Fatahi et al., 2022). How to regulate metabolism while also taking joint health into account is an urgent clinical challenge. Appropriate intensity, reasonable nutritional supplementation, and comprehensive adherence management may become a new direction for comprehensive intervention in this population. Based on the design concept of a randomized controlled trial, this study evaluated the effects of chitosan-assisted moderate-intensity exercise on body weight, fat distribution, metabolic indicators, and lower-limb biomechanical characteristics. To enhance interpretability between metabolic management and functional improvements, we adopted a multi-indicator assessment across body composition, glucose–lipid profiles, and joint mechanics. We hypothesized that, compared with an exercise-only program and with usual lifestyle, adding chitosan would produce greater improvements in metabolic regulation and reductions in lower-limb mechanical load over 12 weeks. The primary outcomes were the changes from baseline to Week 12 in fasting blood glucose and the homeostasis model assessment of insulin resistance (HOMA-IR). The goal of this study is to provide a safer, more feasible, and more effective comprehensive intervention plan for overweight individuals and, through systematic testing of body composition, blood glucose and lipid parameters, as well as joint mechanical indicators, to offer new evidence-based support for the application of polysaccharide-assisted exercise interventions in different age groups or in populations with specific comorbidities.

## 2 Materials and methods

This study was a single-center, parallel-group, open-label randomized controlled trial (RCT) conducted at Zhejiang Industry Polytechnic College from March to December 2024. The protocol was approved by the Ethics Committee of Zhejiang Industry Polytechnic College, and all participants provided written informed consent. Participants and study staff were not blinded to group allocation.

### 2.1 Study subjects

Inclusion criteria: ① Age 18–50 years, no restriction on gender; ② Body mass index (BMI) of 24.0–27.9 kg/m^2^; ③ No systematic weight loss or continuous exercise intervention in the last 3 months; ④ No use of medications affecting blood glucose or lipids in the last 3 months; ⑤ Normal communication ability, voluntarily participated in this study, and signed the informed consent form.

Exclusion criteria: ① Diagnosed by a professional medical institution with type 2 diabetes mellitus and currently receiving hypoglycemic therapy; ② Diagnosed with severe cardiovascular disease, liver or kidney dysfunction, or other severe diseases that may affect the intervention outcome; ③ Significant structural or functional impairments in the lower limbs, including severe arthritis or joint deformity; ④ Pregnant or lactating women; ⑤ Allergic to chitosan preparations or experiencing severe discomfort after taking them; ⑥ Lack of intervention compliance or inability to complete follow-up during the study.

Sample size estimation and random grouping: According to the effect sizes of polysaccharide intervention and exercise intervention on fasting blood glucose and insulin resistance index (HOMA-IR) reported in previous literature, with a significance level α = 0.05 (two-sided) and a test power of 80%, formula calculations indicated that each group required 18 subjects. To reduce the impact of dropouts during the study, 20 subjects were actually included in each group, yielding a total sample size of 60. After all eligible subjects completed baseline assessments, they were randomly assigned at a 1:1:1 ratio by a computer-generated random number sequence, which placed the 60 subjects into three groups: polysaccharide + exercise group (20 subjects), exercise-only group (20 subjects), and control group (20 subjects).

### 2.2 Intervention plan

#### 2.2.1 Polysaccharide intervention

Polysaccharides were administered as an oral chitosan preparation (manufacturer: Carapoly Biotechnology Co.; 500 mg per capsule). The product was a standardized, food-grade chitosan supplied with a manufacturer-issued certificate of analysis (CoA) for the lot used in this trial, confirming identity, chitosan content and degree of deacetylation, the absence of other active polysaccharides, and compliance with microbiological and heavy-metal specifications. Only inert excipients were included. Participants in the polysaccharide + exercise group took two capsules 30 min before breakfast, lunch, and dinner (total daily dose 3,000 mg) for 12 weeks (Rondanelli et al., 2023; Fatahi et al., 2022). Participants in the exercise-only group and the control group did not receive chitosan or placebo.

#### 2.2.2 Exercise prescription

Throughout the 12-week intervention, both the chitosan + exercise and the exercise-only groups followed the same home-based, unsupervised moderate-intensity aerobic program: brisk walking or light jogging for 30–40 min per session, 5 days per week. Exercise intensity was remotely monitored in real time with a wrist-worn heart-rate monitor (Polar M430, Polar Electro Oy), with the target heart rate set at 64%–76% of age-predicted HRmax (HRmax = 208–0.7 × age). After each session, heart-rate data were synchronized to the study management platform; the research team reviewed duration and intensity weekly and contacted participants to troubleshoot deviations. Adherence was defined *a priori* as the percentage of the 60 prescribed sessions completed with (i) duration ≥30 min and (ii) mean HR within the target zone or ≥20 min time-in-zone; participants achieving ≥80% were classified as adherent. To support adherence, participants received standardized instruction at baseline and weekly check-ins (telephone or in person) with individualized feedback; no financial incentives were provided. The control group maintained their usual lifestyle without additional exercise or dietary interventions.

### 2.3 Evaluation indicators and testing methods

A 12-week intervention period was set for this study. The collection time points and methods for each indicator were at baseline (Week 0, denoted as T0), Week 6 (T6), and Week 12 (T12).

#### 2.3.1 Basic information and body composition

The measurement items included height, weight, waist circumference, hip circumference, total and regional fat mass, and fat-free mass. The measurement process was as follows: ① On the measurement day, subjects maintained a fasting state and emptied their bladder before measuring height and weight. The same calibrated medical electronic scale was used to measure weight, recorded to two decimal places. ② The same fixed measuring rod was used to measure height, recorded to two decimal places. ③ A horizontal soft tape was used to measure waist circumference and hip circumference, ensuring the tape was close to the body without compression, and recorded to one decimal place respectively (National Health and Nutrition Examination Survey U.S. and National Center for Health Statistics U.S., 2021a). ④ A dual-energy X-ray absorptiometry instrument (Lunar Prodigy, GE Healthcare, Waukesha, WI, United States) was used to assess total and regional fat mass and fat-free mass; the instrument was calibrated before measurement, and a uniform testing posture was adopted. The measurement results were then recorded (National Health and Nutrition Examination Survey U.S. and National Center for Health Statistics U.S., 2021b). ⑤ Body mass index (BMI) was calculated as weight (kg) ÷ [height (m)^2^].

#### 2.3.2 Metabolic indicators

Collection time points are T0, T6, and T12. After a 10-h overnight fast at each time point, 5 mL of venous blood was collected from the subjects. The detection items and methods are: ① Use an automatic biochemical analyzer AU5800 (Beckman Coulter, Brea, CA, United States) to measure fasting blood glucose, total cholesterol, triglycerides, low-density lipoprotein, and high-density lipoprotein. ② Use high-performance liquid chromatography (Variant II, Bio-Rad Laboratories, Hercules, CA, United States) to determine glycated hemoglobin (HbA1c). ③ Use chemiluminescence immunoassay (KHB 1800, Shanghai Kehua Bio-engineering Co., Ltd.) to measure fasting insulin levels. ④ Calculate the insulin resistance index (HOMA-IR) = [fasting blood glucose (mmol/L) × fasting insulin (µU/mL)] ÷ 22.5.

#### 2.3.3 Lower limb biomechanical characteristics

Collection time points are T0 and T12. The measurement equipment includes the Pedar-X plantar pressure analysis system (Novel GmbH, Munich, Germany) and a Kistler force platform (Kistler Group, Winterthur, Switzerland). The measurement process is as follows: ① Before testing, subjects perform a 5-min simple warm-up, mainly joint mobility exercises and slow walking. ② Subjects wear flat-soled sports shoes and walk across the plantar pressure measurement pad at their usual walking speed. Each subject walks continuously for 5 steps, and the valid data of the middle 3 steps are taken as the average. ③ The plantar pressure analysis system automatically records the maximum plantar pressure, pressure center trajectory, contact area, and contact time. ④ The Kistler force platform is used to record the ground reaction force curve, and Bioware software is used to calculate the peak torque of the knee and ankle joints during the support phase. ⑤ The entire testing process is carried out in the same laboratory environment, with the room temperature controlled at 22–25 °C, ensuring the floor is non-slip and free from other interference.

### 2.4 Intervention adherence and safety assessment

The polysaccharide + exercise group and the control group completed a daily log; participants in the polysaccharide + exercise group recorded whether the study preparation was taken 30 min before each of the three meals, while the control group recorded no-supplement intake for comparability. The research team verified logs weekly (on-site or by telephone) and calculated medication adherence as the number of on-schedule doses divided by the total prescribed doses (3 per day × 84 days), expressed as a percentage; participants achieving ≥80% were classified as adherent. The polysaccharide + exercise and exercise-only groups wore wrist heart-rate monitors to record session duration, maximum heart rate, and average heart rate. A session counted toward the prescription if duration ≥30 min and the mean heart rate was within the 64%–76% HRmax target zone or if ≥20 min time-in-zone was achieved. Exercise adherence was defined as adherent sessions divided by the 60 prescribed sessions, expressed as a percentage; participants achieving ≥80% were classified as adherent. The research team summarized heart-rate data weekly to confirm compliance and contacted participants to address deviations.

Adverse events (AEs; e.g., gastrointestinal discomfort, lower-limb joint pain) were actively queried at each follow-up and testing visit and documented (onset, duration, severity). Pre-specified stopping rules were applied as follows: ①Immediate discontinuation and medical evaluation for any serious adverse event or suspected hypersensitivity (e.g., generalized rash, angioedema, wheeze). ②Temporary hold of chitosan for moderate or worse gastrointestinal symptoms not resolving with supportive measures and persisting >72 h; re-challenge only after clinician clearance; permanent discontinuation if symptoms recur. ③Temporary suspension of exercise for new/worsening musculoskeletal pain or injury that limits safe ambulation/jogging for >48 h, or for cardiorespiratory warning signs (e.g., chest pain, syncope, unexplained dyspnea); resumption only after clinical assessment. ④Pregnancy or participant request prompted withdrawal from the intervention. All AE management decisions (continue, modify, or discontinue) were made by the study physician in consultation with the principal investigator, and participants could withdraw at any time without penalty.

### 2.5 Statistical analysis

All data were analyzed using SPSS Statistics 26.0. Normality was assessed with the Shapiro–Wilk test; normally distributed continuous variables are presented as mean ± SD, and non-normal variables as median (IQR). For between-group comparisons at single time points, one-way ANOVA was used for normal data; non-normal data were log-transformed, or analyzed with non-parametric alternatives as needed. Pre-/post-intervention comparisons employed repeated-measures ANOVA (with Bonferroni-adjusted post-hoc pairwise tests) and paired t-tests when appropriate. Categorical variables (e.g., sex distribution, adverse-event incidence) were compared using the chi-square test or Fisher’s exact test (when expected counts were small). Missing data were handled using a complete-case approach (no imputation); the analysis set included participants with available measurements at the relevant time points. Given prior evidence that moderate-intensity exercise alone improves weight and metabolic indices (Pablo et al., 2022), our primary inferential focus was on “chitosan + exercise vs. exercise-only” and “chitosan + exercise vs. control”. All tests were two-sided with P < 0.05 considered statistically significant.

## 3 Results

### 3.1 Baseline general information of subjects

A statistical analysis was conducted on the baseline indicators of the three groups of subjects, including age, sex, BMI, waist circumference, hip circumference, fasting blood glucose, fasting insulin, HOMA-IR, and blood lipids. According to one-way analysis of variance or chi-square test, there were no statistically significant differences in any of these indicators among the three groups (P > 0.05), suggesting that the three groups had good comparability before the intervention and that the random grouping was appropriate (Table 1).

**TABLE 1:** Baseline general information of study subjects.

Indicator	Polysaccharide + exercise group (n = 20)	Exercise-only group (n = 20)	Control group (n = 20)	Test value	p value
Age (years)	36.40 ± 5.14	37.52 ± 4.60	35.68 ± 5.32	F = 0.463	0.632
Sex (male/female, number)	9/11	8/12	10/10	χ^2^ = 1.548	0.461
BMI (kg/m^2^)	26.10 ± 1.05	25.87 ± 1.20	26.25 ± 1.15	F = 0.713	0.493
Waist circumference (cm)	89.70 ± 5.83	90.45 ± 5.92	88.56 ± 6.43	F = 0.542	0.584
Hip circumference (cm)	99.30 ± 5.03	98.42 ± 4.85	99.15 ± 5.27	F = 0.624	0.539
Fasting blood glucose (mmol/L)	5.56 ± 0.24	5.62 ± 0.28	5.59 ± 0.26	F = 0.336	0.716
Fasting insulin (µU/mL)	10.58 ± 1.32	11.05 ± 1.26	10.72 ± 1.38	F = 0.586	0.560
HOMA-IR	2.72 ± 0.29	2.70 ± 0.28	2.66 ± 0.26	F = 0.248	0.782
Total cholesterol (mmol/L)	5.12 ± 0.28	5.14 ± 0.30	5.10 ± 0.29	F = 0.750	0.477
Triglycerides (mmol/L)	1.52 ± 0.23	1.54 ± 0.20	1.50 ± 0.21	F = 0.318	0.729
LDL (mmol/L)	2.94 ± 0.25	2.96 ± 0.22	2.91 ± 0.24	F = 0.304	0.739
HDL (mmol/L)	1.23 ± 0.11	1.21 ± 0.10	1.22 ± 0.12	F = 0.406	0.668

### 3.2 Changes in body composition

**FIGURE 1:**
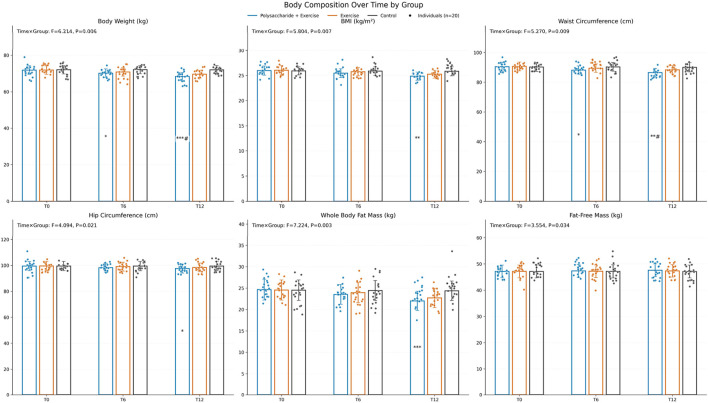
Changes in body composition before and after the intervention. Pairwise comparisons were conducted with Bonferroni correction, “*” indicates comparison with Control, “#” indicates comparison with the exercise-only group (*P < 0.05, **P < 0.01, ***P < 0.001, #P < 0.05, ##P < 0.01, ###P < 0.001).

Body composition. According to repeated-measures ANOVA, the time × group interaction was significant for body weight (F = 6.214, P = 0.006, ηp^2^ ≈ 0.18), BMI (F = 5.804, P = 0.007, ηp^2^ ≈ 0.17), waist circumference (F = 5.270, P = 0.009, ηp^2^ ≈ 0.16), hip circumference (F = 4.094, P = 0.021, ηp^2^ ≈ 0.13), whole-body fat mass (F = 7.224, P = 0.003, ηp^2^ ≈ 0.20), and fat-free mass (F = 3.554, P = 0.034, ηp^2^ ≈ 0.11). After Bonferroni-corrected post-hoc tests, at T12 the polysaccharide + exercise group showed significantly greater improvements in body weight and waist circumference than both the exercise-only and control groups (P < 0.05). For BMI, hip circumference, and total body fat mass at T12, the polysaccharide + exercise group was significantly improved only versus the control group (P < 0.05) (Figure 1).

### 3.3 Changes in metabolic indicators

**FIGURE 2:**
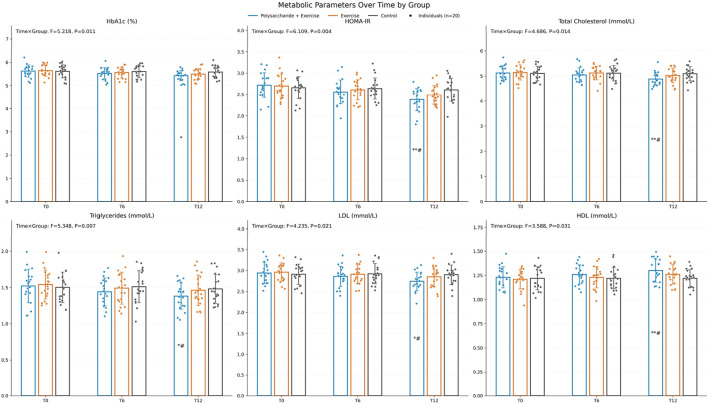
Changes in metabolic indicators before and after the intervention. Pairwise comparisons were conducted with Bonferroni correction, “*” indicates comparison with Control, “#” indicates comparison with the exercise-only group (*P < 0.05, **P < 0.01, ***P < 0.001, #P < 0.05, ##P < 0.01, ###P < 0.001).

Metabolic parameters. The time × group interaction was significant for all metabolic indicators: HbA1c (F = 5.218, P = 0.011, ηp^2^ ≈ 0.15), HOMA-IR (F = 6.109, P = 0.004, ηp^2^ ≈ 0.18), total cholesterol (F = 4.686, P = 0.014, ηp^2^ ≈ 0.14), triglycerides (F = 5.348, P = 0.007, ηp^2^ ≈ 0.16), LDL-C (F = 4.235, P = 0.021, ηp^2^ ≈ 0.13), and HDL-C (F = 3.588, P = 0.031, ηp^2^ ≈ 0.11). After Bonferroni correction, all metabolic indicators except HbA1c improved significantly more in the polysaccharide + exercise group than in both comparison groups at T12 (all P < 0.05) (Figure 2).

### 3.4 Changes in lower limb biomechanical characteristics

**FIGURE 3:**
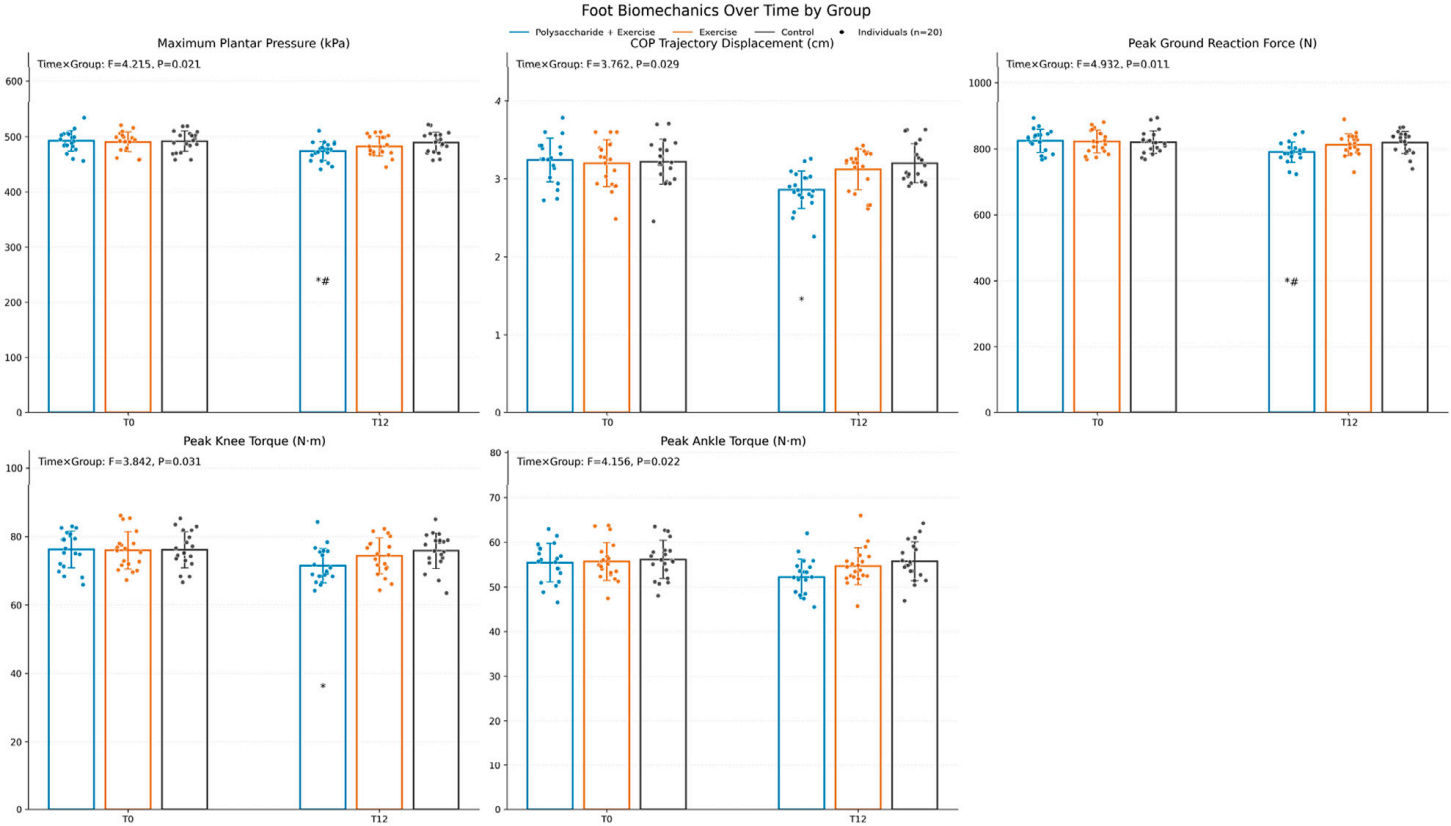
Changes in lower limb biomechanical indicators before and after the intervention. Pairwise comparisons were conducted with Bonferroni correction, “*” indicates comparison with Control, “#” indicates comparison with the exercise-only group (*P < 0.05, **P < 0.01, ***P < 0.001, #P < 0.05, ##P < 0.01, ###P < 0.001).

Lower-limb biomechanics. The time × group interaction was significant for maximum plantar pressure (F = 4.215, P = 0.021, ηp^2^ ≈ 0.13), center-of-pressure trajectory displacement (F = 3.762, P = 0.029, ηp^2^ ≈ 0.12), peak ground reaction force (F = 4.932, P = 0.011, ηp^2^ ≈ 0.15), and peak torques of the knee (F = 3.842, P = 0.031, ηp^2^ ≈ 0.12) and ankle (F = 4.156, P = 0.022, ηp^2^ ≈ 0.13). After Bonferroni-corrected pairwise comparisons, the polysaccharide + exercise group exhibited significantly lower maximum plantar pressure and peak ground reaction force at 12 weeks compared with both the exercise-only and control groups (P < 0.05) (Figure 3).

### 3.5 Intervention adherence and adverse event statistics

Independent samples t-test showed a statistically significant difference in intervention adherence rates between the polysaccharide + exercise group and the exercise-only group (P = 0.002), with the former having a higher adherence rate. There was no statistically significant difference in the incidence of adverse events among the three groups (χ^2^ test) (P > 0.05), and all adverse events were mild or moderate, with no severe adverse events reported (Table 2).

**TABLE 2:** Intervention adherence and adverse event statistics.

Indicator	Polysaccharide + exercise group	Exercise-only group	Control group	Test value	p value
Intervention adherence rate (%)	88.42 ± 4.14	83.68 ± 4.76	—	t = 3.360	0.002
Total number of adverse events (cases, %)	4 (20.0)	3 (15.0)	2 (10.0)	χ^2^ = 1.232	0.561
Gastrointestinal discomfort (cases, %)	1 (5.0)	1 (5.0)	0 (0.0)	χ^2^ = 1.146	0.564
Joint pain (cases, %)	2 (10.0)	1 (5.0)	1 (5.0)	χ^2^ = 2.394	0.302
Other (cases, %)	1 (5.0)	1 (5.0)	1 (5.0)	χ^2^ = 0.000	1.000
Mild (cases, %)	3 (15.0)	2 (10.0)	1 (5.0)	χ^2^ = 1.864	0.394
Moderate (cases, %)	1 (5.0)	1 (5.0)	1 (5.0)	χ^2^ = 0.000	1.000
Severe (cases, %)	0 (0.0)	0 (0.0)	0 (0.0)	—	—

## 4 Discussion

This study shows that after a 12-week intervention, the combination of polysaccharides and moderate-intensity exercise led to a more pronounced decrease in body weight, BMI, waist circumference, hip circumference, and total body fat mass in overweight individuals. This result suggests that although exercise alone can improve body composition, the auxiliary role of polysaccharides may further enhance fat metabolism efficiency, thereby accelerating reductions in body fat and circumference. Chitosan and other polysaccharides in the gastrointestinal tract may inhibit the absorption of some fats and improve the body’s metabolic status by influencing gut flora and intestinal barrier function (Claire et al., 2022; Antonella et al., 2021). Moderate-intensity exercise plays a crucial role in negative energy balance and muscle energy metabolism, increasing energy consumption and activating aerobic metabolic pathways (Singh et al., 2021). On this basis, when both interventions occur simultaneously, the processes of fat breakdown and consumption may undergo a higher level of synergistic enhancement. Compared with exercise alone or not using polysaccharides, the dual intervention of polysaccharides plus exercise can more effectively alter the progression of abnormal body composition in overweight populations, which is of positive significance for weight management and may help prevent obesity-related chronic diseases. Previous research examining the combination of seaweed polysaccharides or soluble dietary fiber and exercise has also confirmed its positive effects on body fat control and circumference management (Liu et al., 2021), but the specific mechanisms still need further elucidation at the experimental and molecular levels. By integrating multiple indicators such as BMI, waist-hip circumference, and body fat mass, this study has, to some extent, demonstrated that polysaccharides can be incorporated as an auxiliary strategy into daily exercise prescriptions. In the future, conducting larger-scale studies among different age groups, varying degrees of obesity, or populations with comorbid metabolic diseases would be more conducive to clarifying the target groups and optimal intervention windows for this combined intervention model, potentially providing more practical and accessible intervention programs for clinical practice and public health.

This study found that, after combining polysaccharides with moderate-intensity exercise, there was a more pronounced improvement in indicators such as blood glucose, blood lipids, and HOMA-IR compared to the control group and the exercise-only group, suggesting that this composite intervention model has positive significance for enhancing the body’s metabolic regulation. These changes were statistically significant: the time × group interaction was significant for HOMA-IR (F = 6.109, P = 0.004), triglycerides (TG) (F = 5.348, P = 0.007), and LDL-C (F = 4.235, P = 0.021), with Bonferroni-corrected post-hoc tests showing greater improvements at T12 in the chitosan-plus-exercise group than in both comparison groups (all P < 0.05). Polysaccharides may bind cholesterol and free fatty acids in the gastrointestinal tract, thereby reducing lipid absorption and blood lipid formation, while to some extent improving insulin sensitivity and thus alleviating insulin resistance (Chen et al., 2021). Moderate-intensity exercise brings a dual optimization of blood glucose and blood lipid levels by increasing energy expenditure, promoting mitochondrial oxidation, and enhancing the uptake and utilization of glucose and lipids by skeletal muscle (Wang et al., 2020). In addition, only glycated hemoglobin did not show obvious improvement after the intervention, which may be related to the relatively limited intervention duration or the fact that baseline glucose metabolism abnormalities in the subjects were not yet severe. This pattern is consistent with the biology of HbA1c, which reflects average glycemia over approximately 2–3 months and may lag behind shorter-term improvements (American Diabetes Association Professional Practice Committee et al., 2024). It also indicates that changes in long-term cumulative indicators of blood glucose may require a longer period to fully manifest (Molina-Garcia et al., 2019). From the perspective of preventing and slowing the progression of metabolic syndrome in overweight populations, the study results emphasize that a combined strategy of polysaccharides and exercise may provide a feasible option for early intervention. No severe adverse events were observed during the entire intervention, indicating that the safety and operability of this program are relatively assured in practical application. In the future, more centers and longer follow-up are needed to further explore the lasting effects of polysaccharides in maintaining stable blood glucose, reducing insulin resistance, and lowering blood lipids. More in-depth molecular and clinical research will also be necessary to clarify the mechanisms of action, thus providing a more solid evidence base for clinical application and health management.

Polysaccharide-assisted exercise has been further confirmed in this study to have a positive impact on lower limb biomechanical characteristics, with the polysaccharide + exercise group showing the most significant reduction in maximum plantar pressure, peak ground reaction force, and peak knee-ankle joint moments after the intervention. The decrease in body weight directly reduces lower limb loading, which is particularly evident in terms of foot-bearing pressure; the simultaneous reductions in maximum plantar pressure and peak ground reaction force indicate that exercise impact and joint stress have been alleviated to some extent. The decrease in total body fat mass reduces the mechanical load on joint cartilage, helping to optimize the distribution of lower limb force lines (Nagaoka et al., 2019). Meanwhile, the improvements in muscle strength and endurance brought about by the combined intervention of polysaccharides and moderate-intensity exercise enable better muscular support and neuromuscular control for the major joints such as the knee and ankle when bearing weight, making the gait more stable and thereby lowering joint moments. This further highlights the synergistic effect between reduced body fat and enhanced muscle function (Silva et al., 2018). Unlike exercise alone or dietary intervention alone, this polysaccharide-assisted approach not only promotes a negative energy balance but also helps some subjects maintain their fat-free mass during sustained exercise, preventing substantial muscle loss (Chen et al., 2024). When the joints are in motion and have better muscular protection, the areas of pressure concentration under the foot and in the joints can be dispersed, thereby reducing the potential risk of exercise-related injuries or chronic joint degeneration. For overweight or mildly obese populations, this composite intervention that combines weight management with a basic exercise prescription is of practical significance. The direction of these changes is consistent with current physical activity and weight-management guidance, which recommends at least 150 min per week of moderate-intensity aerobic activity alongside multicomponent programs that include muscle-strengthening and balance/neuromotor training; our findings extend these recommendations by suggesting that pairing such aerobic exercise with chitosan may further lower mechanical loading and improve functional indices. Under conditions of adequate resources and higher adherence, incorporating strength or balance training into a polysaccharide-assisted system may yield even more significant improvements in exercise function. However, this should be framed as a hypothesis for future, adequately powered trials rather than as a conclusion of the present study. In clinical practice, corresponding polysaccharide and exercise prescriptions can be matched to population characteristics and exercise preferences, striving to enhance safety and adherence through individualized design, and ultimately form an effective plan for weight reduction and joint function maintenance. As the sample size expands and follow-up extends over a longer period, the value of this composite intervention in preventing lower limb osteoarticular lesions and preserving daily physical activity will be further evidenced, providing more feasible options for patients with obesity combined with joint problems.

The primary limitations of this study include the relatively small sample size, short follow-up duration of 12 weeks, and open-label trial design without placebo capsules for the exercise-only and control groups. The absence of blinding and placebo control may introduce performance and expectancy bias, such as behavioral changes or varying adherence, potentially influencing between-group comparisons despite most outcomes being objectively assessed (e.g., DXA, automated biochemical assays, and biomechanical measurements). Free-living physical activity outside the intervention sessions was not monitored, leaving the possibility that unmeasured incidental activity contributed to greater improvements in the polysaccharide-plus-exercise group. Future studies should incorporate accelerometry or step tracking to account for habitual activity levels. Multicenter trials with participant and assessor blinding, use of appearance-matched placebos, and extended follow-up periods would enhance internal validity and the generalizability of the findings. Although the positive effects of combining polysaccharides with exercise on metabolic and lower limb biomechanical indicators in overweight populations were preliminarily observed, it remains uncertain whether these effects can be sustained over the long term. In particular, some long-term indicators related to metabolic syndrome, such as glycated hemoglobin, did not show significant improvement in the short term, suggesting that future research should consider a longer follow-up period to determine the long-term metabolic benefits and sustainability of polysaccharide-assisted exercise. Although the overall intervention adherence rate was relatively high, there may be a risk of declining adherence in actual promotion and application, especially among community populations lacking effective supervision methods. This highlights the need to further explore specific strategies to improve long-term adherence. Potential solutions include wearable- or app-based remote monitoring with automated reminders and individualized feedback, brief telecoaching contacts, and community-based group programs to provide peer support and accountability. In addition, this study only included overweight adults, and future research should be expanded to include obese or clinically patient populations with significant metabolic abnormalities to evaluate its clinical generalizability. We focused on overweight rather than obesity to test early-intervention feasibility while limiting musculoskeletal loading and injury risk during moderate-intensity activity and to minimize confounding from anti-obesity pharmacotherapy or mobility limitations; the trial was not powered for age- or sex-stratified analyses, so applicability to older adults and sex-specific responses should be confirmed in larger studies. Regarding lower limb biomechanical assessment, this study was limited to basic gait analysis. In the future, dynamic exercise testing or joint imaging examinations could be added to more comprehensively elucidate the protective effects of polysaccharide-assisted exercise on joint structure and function. Furthermore, this study did not involve testing of the gut microbiota or metabolomics related to polysaccharides. Based on current evidence, we hypothesize that the combined intervention may act through microbiome-mediated pathways—such as increased short-chain fatty acid production, improved intestinal barrier integrity with reduced endotoxemia, and altered bile-acid signaling—thereby contributing to the observed improvements in insulin resistance and lipid profiles; future work should incorporate fecal metagenomics/metabolomics and SCFA/bile-acid profiling to test these mechanisms (Zhang et al., 2022).

## 5 Conclusion

The results of this study indicate that an intervention model combining polysaccharides with moderate-intensity exercise can significantly improve body composition, metabolic indicators, and lower limb biomechanical characteristics in overweight populations, and demonstrates better effects than exercise alone or the control group in reducing body weight, improving insulin resistance and blood lipid levels, and alleviating plantar and joint load. Polysaccharides such as chitosan, synergistically combined with regular exercise, can effectively promote fat metabolism, improve metabolic health, and exhibit good safety and adherence, suggesting that this composite intervention program could serve as one of the effective strategies for managing overweight individuals.

## Data Availability

The original contributions presented in the study are included in the article/supplementary material, further inquiries can be directed to the corresponding author.
